# Distribution, Sources and Risk Assessment of Polychlorinated Biphenyls in Soils from the Midway Atoll, North Pacific Ocean

**DOI:** 10.1371/journal.pone.0071521

**Published:** 2013-08-08

**Authors:** Jing Ge, Lee Ann Woodward, Qing X. Li, Jun Wang

**Affiliations:** 1 Key Laboratory of Aquatic Botany and Watershed Ecology, Wuhan Botanical Garden, Chinese Academy of Sciences, Wuhan, China; 2 U.S. Fish and Wildlife Service, Pacific Reefs NWRC, Honolulu, Hawaii, United States of America; 3 Department of Molecular Biosciences and Bioengineering, University of *Hawaii* at Manoa, Honolulu, Hawaii, United States of America; National Cancer Institute at Frederick, United States of America

## Abstract

Concentrations of 28 polychlorinated biphenyls (PCBs) were assessed in soils from the Midway Atoll in the central North Pacific Ocean. The analytical procedure involved the application of accelerated solvent extraction (ASE) and gas chromatography coupled with ion trap mass spectrometric detection (GC/ITMS) for identification and quantification. Among the 28 PCB congeners studied, 26 of them, except CB195 and CB209, were detected in the analyzed samples at different frequencies. The total concentrations of 28 indicator PCBs (ΣPCBs) ranged from 2.6 to 148.8 ng g^−1^ with an average value of 50.7 ng g^−1^ and median of 39.5 ng g^−1^. Sources and congeners’ pattern of PCB were investigated in the soil of Midway Atoll. The principal component analysis indicated that the compositions of PCBs in most of the soil samples were similar. The total concentrations of PCBs were used to assess the cancer risk probabilities in humans via ingestion, dermal contact and inhalation of soil particles. Very low cancer risk was found in all soil samples caused by ΣPCBs.

## Introduction

Persistent organic pollutants (POPs), as PCBs and OCPs are synthetic compounds with great chemical stability. Due to the wide use throughout the world since the middle of the past century, these compounds are ubiquitous in the environment and pose an environmental and human risk [1], [2]. Some of these pollutants are highly toxic and have a large variety of chronic effects, including endocrine dysfunction, mutagenesis and carcinogenesis. PCBs are hydrophobic and have considerable accumulation potential in organisms and magnification through the food chain [3]. PCBs are components of transformers, capacitors, hydraulic and heat exchange fluids [4], and the dismantling and burning activities promote its leakage into the surrounding soils. PCBs are believed to act as endocrine disruptors that affect hormone regulation [5]. Significant correlations between biochemical parameters (serum hormone concentrations and cytochrome P450 enzyme activities) and residues of endocrine disrupting chemicals were found in some marine animal species, which indicates that these chemicals may impose toxic effects in animals even at the current levels of exposure. In general, water birds and marine mammals accumulated the dioxin-like compounds with much higher concentrations than humans, implying higher risk from exposure in wildlife [6].

The Midway Atoll (178 °W, 28 °N) is in the North Pacific Ocean 1100 miles northwest of Honolulu, Hawaii. The Midway atoll consists of two main islands, Sand and Eastern, surrounded by a fringing coral reef [7]. The Midway Atoll was under the Navy jurisdiction from 1903 to 1996. There are many environment contaminants that resulted from 90 years of military operations. Contaminants included PCBs, polycyclic aromatic hydrocarbons (PAHs), petroleum hydrocarbons, pesticides such as dichlorodiphenyltrichloroethane (DDT) and dichlorodiphenyldichloroethane (DDE), and numerous metals. During World War II, several aircraft carriers and hundreds of aircraft were sunk near the Midway. PCBs have been released from the generators, capacitors, etc. of the sunken aircraft carriers and aircraft. These compounds have been accumulating in the soils and marine life surrounding these sunken vessels.

Even though heavily modified by human activity nearly hundred years, the islands provide breeding and feeding habitats for 17 species of seabirds with an aggregate population of nearly 2 million. The Midway Atoll is also a habitat for threatened green sea turtles and Hawaiian monk seals [8]. In order to limit the exposure by ecological receptors, remedies have been implemented. However, those contaminants are not easily degraded. The bulk of POPs in the environment resides in soils and sediments where they primarily partition into organic matter. Small changes in the mass of soils/sediments would have a major impact on concentrations in ‘adjacent’ media, such as air or water [2]. Many organochlorine POPs have high affinity for soil and are retained in this environment medium for a long time. Such POPs maybe taken by crops or by grazing animals and hence reach the human food chain. They may also be washed in run-off from the land into watercourses.

The objectives of the study were to determine the concentrations of PCBs in the soil of the Midway Atoll, and analyze the potential sources of PCBs in this area. The study also conducted a human health risk assessment on cancer, in order to evaluate the potential carcinogenic risk based on the concentrations of PCBs in soil.

## Materials and Methods

### Study Area and Sample Collection

**Figure 1:**
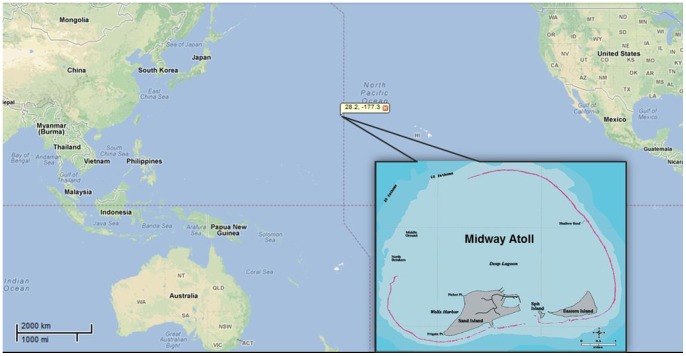
Locations of study area in the Midway Atoll, North Pacific Ocean.

Midway Atoll is located at the northwest end of the Hawaiian Islands archipelago, at 28.208°N latitude and −177.379°W longitude (Fig. 1). This is approximately 2,000 km from Honolulu, Hawaii and 4900 km from Portland, Oregon. The atoll is comprised of two main islands, Sand and Eastern, and one smaller islet, enclosed within a reef approximately 8 km. As part of the Midway Atoll, Sand Island has a long history of use for communications, commercial and military purposes. Midway was a base for military operations between 1941 and the early 1990’s. As such, portions of Sand Island were, and continue to be occupied by an airfield, buildings and other structures to support operations and staff that live on the island. One hundred and eleven soil samples were collected from the Sand and Eastern islands in Midway Atoll, the North Pacific Ocean.

One hundred and eleven surface soil samples (0–15 cm) were collected in 2006 from different station on Midway Atoll. The samples were immediately transferred to the laboratory and frozen at −20°C until processed for analysis. All samples were collected under the permit of the U.S. Fish and Wildlife Service.

### Analytes

**Figure 2:**
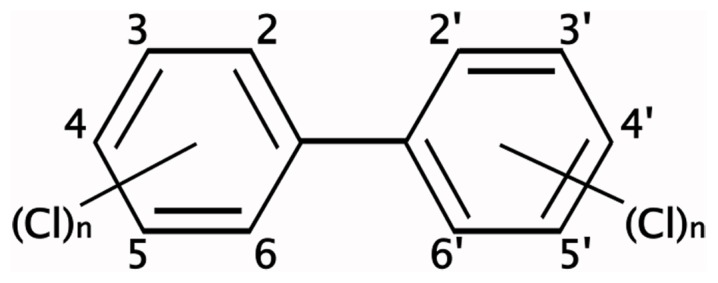
The basic structure of PCBs.

The 28 PCBs indicators were studied including di-CDs (PCB 8), tri-CBs (PCB18, 28), tetra-CBs (PCB 44, 52, 66, 77, 81), penta-CBs (PCB 101, 105, 114, 118, 123, 126), hexa-CBs (PCB 128, 138, 153, 156, 157, 167, 169), hepta-CBs (PCB 170, 180, 187,189), octa-CBs (PCB 195), nona-CBs(PCB 206) and deca-CBs (PCB 209). ^13^C-PCBs 28, 123, 169 and 170 were used as surrogate standards, and pentachloronitrobenzene (PCNB) was used as an internal standard. The basic PCB structure is shown in Fig. 2.

### Sample Preparation, Extraction and Cleanup

Soil samples were freeze-dried for 24 h, pulverized and sieved through 80-mesh stainless steel. The sample cell (about 6–12 g) was loaded into an accelerated solvent extractor (ASE) 200 system (Dionex, Sunnyvale, CA, USA). The extraction was performed with a mixture of acetone and methylene chloride (1∶1, v/v) at a pressure of 1500 psi and temperature of 100°C for three static cycles, a flush volume of 60% of the cell volume and a N_2_ purge time of 5 s. A mixture of Ottawa sand and Na_2_SO_4_ was extracted in the same manner as the sample blank. All samples were extracted in triplicate. After the extract was dried with 30 g of anhydrous sodium sulfate and rinsed with hexane (2 mL), it was concentrated to approximately 2 mL by using a rotary evaporator. The concentrated extract in hexane was cleaned up on an 8 mm i.d. aluminum/silica column. The column was packed, from the bottom to the top, with neutral silica (4 g, 3% deactivated), neutral alumina (2.0 g, 6% deactivated), and anhydrous sodium sulfate (1 cm). The column was eluted with 20 mL of methylene chloride/hexane (1∶1) to yield the PCBs and other POP fractions. The fraction was concentrated to 20 µL under a gentle stream of high purity nitrogen gas after 20 µL of dodecane were added as the trapping solvent. A known quantity of pentachloronitrobenzene (PCNB) was added as an internal standard prior to gas chromatography–ion trap mass spectrometry (GC/ITMS) analysis.

### Gas Chromatography and Ion Trap MS (GC/ITMS) Analysis

The samples were analyzed on a Varian Saturn 2000 (Palo Alto, CA) gas chromatograph with mass spectrometric (ion trap) detection (GC/ITMS). The column was a capillary column DB-5MS (J&W Scientific, Inc., 30 m ×0.25 mm i.d. ×0.25 µm). Helium was used as the carrier gas. The oven temperature started at 100°C for 1 min, increased to 170°C at a rate of 30°C min^−1^ and held for 5 min, increased to 270°C at a rate of 5°C min^−1^ and held for 2 min, and finally increased to 300°C at a rate of 5°C min^−1^ and held for 15 min. The injector temperature was set at 300°C. The detector was 320°C. The injection volume was 2 µL. The injection mode was splitless. The purge time was 1.2 min. The ion trap temperature was 200°C, manifold 80°C, and the transfer line 210°C. The determination was performed by using selected ion monitoring (SIM) mode. Concentrations were calculated from external standards with the MS.

### Quality Assurance and Quality Control (QA/QC)

Average PCB recoveries and relative standard deviations (RSDs) were first obtained to evaluate the method performance by multiple analyses of 10 replicate spiked soil samples with a concentration of 5 ng g^−1^ for each PCB congener. The extraction and cleanup methods according to the procedure described above. A solvent blank and matrix blank were processed through the entire procedure and analyzed prior to and after every 10 samples. Working standard solutions of PCBs were run at the beginning of sample analysis to determine the relative response factors and evaluate peak resolution. Each sample was analyzed in triplicate unless otherwise stated. The limit of detection (LOD) was determined as signal-to-noise ratio of 3∶1. Ranges of average PCB recoveries and RSDs in 10 replicate spiked soil samples were from 78% to 104% and 10% to 15%, respectively. LODs for PCBs ranged from 1–50 pg g^−1^ dependent upon the degree of chlorination of different congeners and were approximately 5 pg g^−1^ for most congeners. The average recoveries of the surrogate standard ^13^C-PCBs 28, 123, 169 and 170 ranged from 72% to 86%. Reported PCB concentrations were not corrected according to the recoveries of the surrogate.

### Statistical Analysis

Principal component analysis (PCA) was carried out in SPSS for the available samples to identify the possible source of PCBs and the distribution of PCBs congeners in Midway Atoll. The PCA component matrix was rotated using a Varimax rotation to the axes, which maximized the variance of the components.

### Cancer Risk Assessment

Cancer risks via ingestion, dermal contact and inhalation of soil particles were estimated on the following *Eqs. (1), (2)* and *(3)*, which were adapted from two documents from the U.S. Environmental Protection Agency (U.S. EPA) [9], [10].
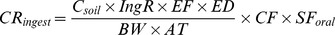
(1)where CR_ingest_ is the cancer risk via accidental ingestion of soil, C_soil_ is the concentration of the contaminant in soil (mg kg^−1^), IngR is the ingestion rate of soil (mg d^−1^), EF is the exposure frequency (d a^−1^), ED is the exposure duration (a), BW is the average body weight (kg), AT is the averaging time (d), CF is the conversion factor (1×10^−6^ kg mg^−1^), SF_oral_ is the oral slope factor (2.0E+00 (mg kg^−1^ d^−1^) ^−1^).
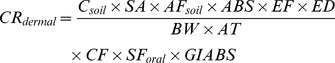
(2)where CR_dermal_ is the cancer risk via dermal contact of soil, SA is the surface area of the skin that contacts the soil (cm2), AF_soil_ is the skin adherence factor for soil (mg cm2), ABS is the dermal absorption factor (0.1). GIABS is the fraction of contaminant absorbed in gastrointestinal tract.
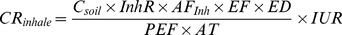
(3)where CRinhale is the cancer risk via inhalation of soil, InhR is the inhalation rate (m3 d−1), AFInh is the absorption factor for the lungs, PEF is the particle emission factor (1.36 × 109 m3 kg−1), IUR is the inhalation unit risk (5.7E-01 (mg m3) −1), which equals to the slope factor via inhalation. The PEF concerns the inhalation of pollutants adsorbed to inhalable particles (PM10).

The estimation of cancer risks via ingestion, dermal contact and inhalation was based on a human lifespan of 70 years. Soil ingestion rate (IngR) of 100 mg d^−1^ was used in this case for adults. Exposure duration (ED) of 70 years was used based on the average lifespan, and an assumed exposure frequency (EF) of 350 days/year, excluding 15 days of holidays, was adopted. An upper-bound value of averaging time (AT) was calculated as 70 × 365 = 25,550 days. A body weight of 70 kg was selected according to the local situation, in which the contact surface area of skin with soil was set at 3300 cm^2^, assuming that hands and arms were exposed. AF_soil_ was 0.2 mg cm^2^. Inhalation rate was 15.8 m^3^ d^−1^ for adults. The values of GIABS and AF_Inh_ were set at 1 for preliminary risk assessment.

According to the Human Health Evaluation Manual [11], cancer risk can increase for the same individuals by exposing in different exposure pathways. Hence, the estimation of total cancer risk through ingestion, dermal contact and inhalation was calculated by adding the results of *Eqs. (1), (2)* and *(3)*. The following qualitative ranking of cancer risk estimates was used to rank the risk from very low to very high: very low (value ≤10^−6^); low (10^−6^< value ≤10^−4^); moderate (10^−4^< value ≤10^−3^); high (10^−3^≤ value <10^−1^); and very high (value ≥10^−1^) [12].

## Results and Discussion

### Total Concentrations and Congener Profiles of PCBs

The 28 PCBs indicators including PCB 8, 18, 28, 44, 52, 66, 81, 77, 101, 105, 114, 118, 123, 126, 128, 138, 153, 156, 157, 167, 169, 170, 180, 187, 189, 195, 206 and 209 were used to evaluate the contamination status of PCBs in the Midway Atoll area. The concentrations of individual PCB congeners in soils were in the range from non-detectable to 62.77 ng g^−1^. Table 1 shows the concentration ranges, average, median and total concentrations. It is noteworthy that the concentration of PCB 77 was much higher than the other congeners. PCB 77 has four chlorines in non-ortho positions, which is very potent mimics of 2,3,7,8-tetrachlorodibenzo-p-dioxin (TCDD) and 2,3,7,8-tetrachlorodibenzofuran (TCDF) both in P-450 induction and toxic effects [13]. Congeners PCB 8, 77, 81, 118, 123, 138, 153, 180 and 187 made the great contribution to the total concentrations. These nine collectively accounted for approximately 77% of the total congener concentration in soils. The congeners, PCB 156, 157, 167, 169, 195 and 206 were detected in no more than 10% of the samples and generally made small (<1%) individual contributions to total loading. The deca-chlorinated congener PCB 209 was not present in any of the samples; PCB-206 was only detected in 3 of the 111 samples, while PCB-195 was only detected in 2 of the samples. The most toxic congeners PCB 126 (TEF = 0.1) and PCB 169 (TEF = 0.01) were found in 58 and 15 out of 111 samples, respectively.

**Table 1:** The concentrations of PCB congeners in soils from the Midway Atoll.

PCB congeners	Concentrations (ng g^−1^)			PCB congeners	Concentrations (ng g^−1^)		
	Range	Average	Median		Range	Average	Median
CB-8	nd-8.78	3.20	3.03	CB-153	nd-10.2	3.13	2.79
CB-18	nd-3.43	0.46	0.28	CB-138	nd-10.4	3.12	2.88
CB-28	nd-3.48	0.58	0.16	CB-128	nd-11.9	0.44	0.00
CB-52	nd-4.12	0.54	0.19	CB-167	nd-1.15	0.06	0.00
CB-44	nd-4.86	0.57	0.37	CB-156	nd-0.70	0.03	0.00
CB-66	nd-7.96	1.40	1.01	CB-157	nd-1.55	0.04	0.00
CB-81	nd-59.5	12.0	1.35	CB-169	nd-2.01	0.12	0.00
CB-77	nd-62.8	14.1	8.27	CB-187	nd-5.32	1.28	1.03
CB-101	nd-3.10	0.91	0.79	CB-180	nd-7.01	2.05	1.74
CB-123	nd-6.66	2.02	1.58	CB-170	nd-2.68	0.37	0.00
CB-118	nd-6.26	1.58	1.29	CB-189	nd-5.14	0.30	0.00
CB-114	nd-2.95	0.46	0.00	CB-195	nd-0.89	0.01	0.00
CB-105	nd-20.6	1.21	0.60	CB-206	nd-1.18	0.03	0.00
CB-126	nd-12.2	0.67	0.19	CB-209	Nd	nd	nd

Note: nd denotes not detected.

The total concentrations of the 28 indicator PCBs (ΣPCBs) ranged from 2.6 to 148.8 ng g^−1^ with an average of 50.7 ng g^−1^ and median of 39.5 ng g^−1^, and the highest value of 148.8 ng g^−1^ was found at B8-5 site. In comparison with other places around the world, the total concentration of PCBs in soils of the Midway Atoll was significantly higher than those in Moscow (2–34 ng g^−1^) [14], Azerbaijan (0.4–0.7 ng g^−1^) [15], e-waste recycling area of Southeast China (0–55 ng g^−1^) [16] and James Ross Island of Antarctica (0.5–1.8 ng g^−1^) [17], and at the same level as those in East coast of Antarctic and France (0.1–150 ng g^−1^) [18], but remarkably lower than those in Poland (80–680 ng g^−1^) [19]. In general, the total concentration of PCBs in the Midway Atoll was at a medium level compared to these areas around the world.

**Figure 3:**
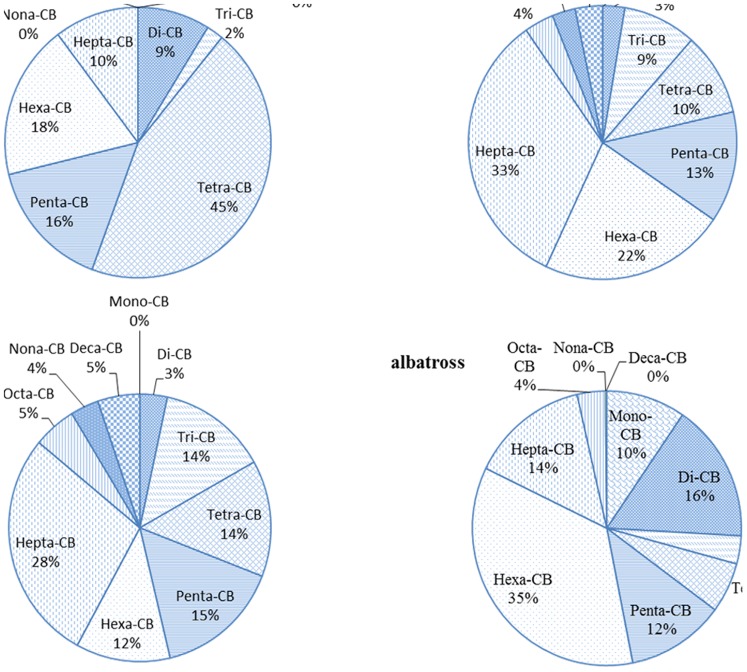
The composition of PCB homologues in soil, black-footed albatross, surface water and marine sediment of the Midway Atoll.

**Figure 4:**
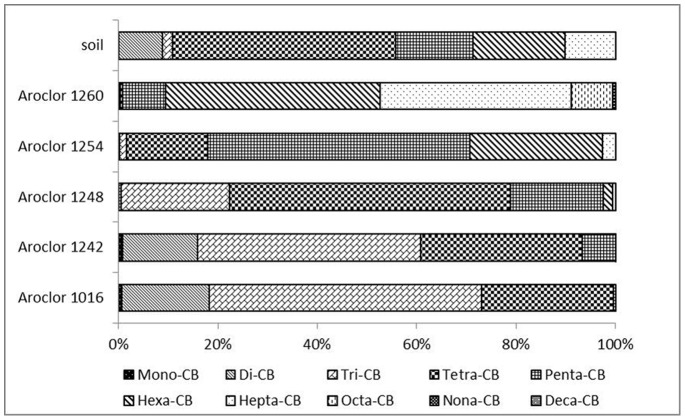
Composition of PCB homologues (average %) in the soil samples and in reference Aroclors 1016, 1242, 1248, 1254 and 1260.

As to the profile of PCBs homologues (Fig. 3.), the dominant congeners were tetra-chlorobiphenyls (tetra-CBs) (mean: 45%), followed by hexa-CBs (mean: 18%) and penta-CBs (mean: 16%), accounting for more than 79% of the total PCBs concentration (Fig. 3). The top three homologues found in all soil samples of the Midway Atoll were low chlorinated congeners including CB-77, CB-81 and CB-8. The pattern of PCBs congeners in the soil of the Midway Atoll was different from that in global background soil which is dominant by hexa-CBs (∼46%) and penta-CBs (∼27%) [20]. Aroclor is a PCB mixture produced from approximately 1930 to 1979, which is a potential source of PCB pollution in the environment. Aroclors 1016, 1242, 1248, 1254 and 1260 were the most widely used among the nine Aroclors [21]. The PCBs profile in the soils obtained did not resemble any of these in the technical mixtures 1016, 1242, 1248, 1254 and 1260 (Fig 4.), which could be attributed to weathering and changes during bioaccumulation. From Fig. 4, we can conclude that there was no Aroclors 1260 used in this area because no octa-CBs was detected in the soil samples. However, the data was not sufficient to draw further conclusions about the original Aroclors of PCBs in the soils of the Midway Atoll. Since Midway Atoll is a tiny island located in the middle of North Pacific Ocean and which was only for military use, moreover, 9000 tons of soil was shipped to this area from Oahu and Guam [8], so PCBs could be transported to this place at the same time. The long-range transmission of low chlorinated congeners from other areas could contribute some of the pollution too.

The composition of PCBs in soil from our study was also compared to the studies of Caccamise et al. [22] and Hope et al. [7] in black-footed albatross and marine sediment in this area. Fig. 3 shows that the PCBs patterns varied largely among different matrices. The results illustrate that penta, hexa and hepta-CBs comprised large proportion in all of the matrices. Hexa-CBs dominated in albatross, while tetra-CBs contributed the most in soils. In the surface water and marine sediment, hepta-CBs made the biggest contributions. Mono-CBs was only found in albatross, while nona- and deca-CBs were only found in marine sediment and water. None of these three was found in soils. Low chlorinated PCBs, like mono-CBs, are more susceptible to losses through volatilization and possibly microbial degradation [23], [24]. While moderately and highly chlorinated PCBs may remain at constant levels in the aquatic environment because they are less volatile, adsorb readily to sediments and are more resistant to microbial degradation [25]. Hexa-CBs are easily accumulated in albatross.

### PCBs Congeners’ Pattern in Soil

**Figure 5:**
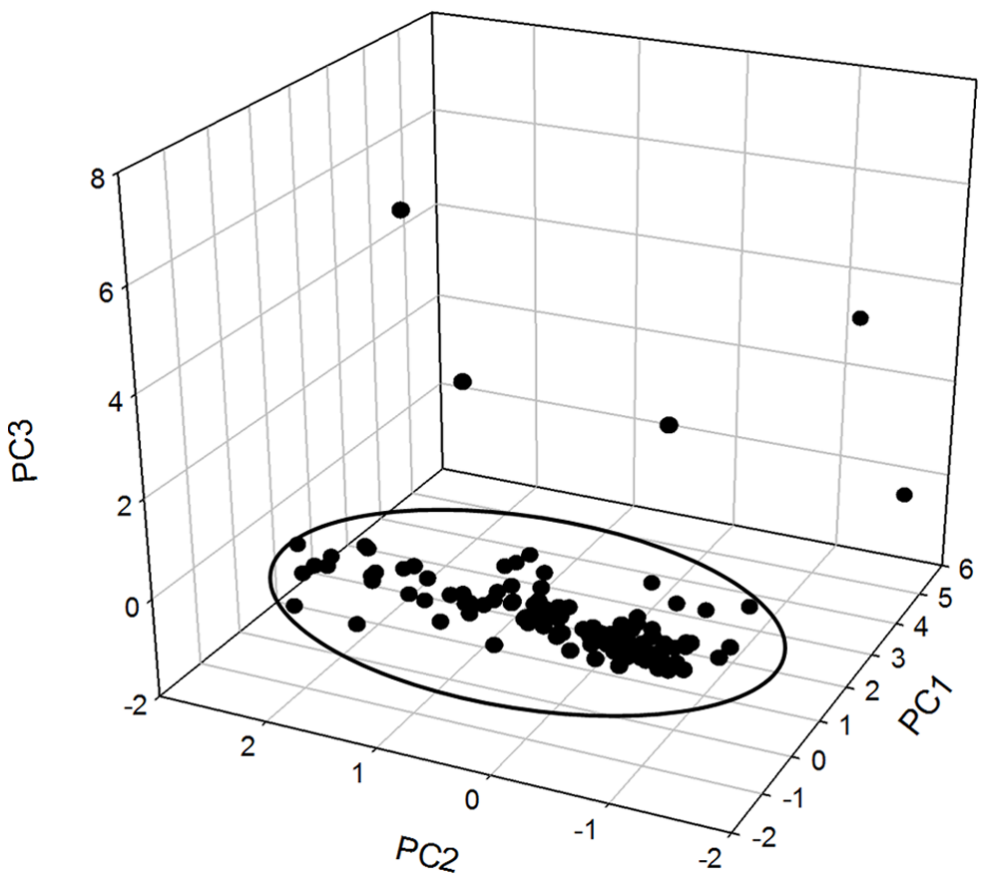
Principal component plot of PCBs from the soil samples of the Midway Atoll.

The patterns and relationships between congeners and sample locations were further investigated using principal component analysis (PCA), performed with SPSS software. PCA describes the statistical relationship between the variables and simplifies the data set converting it into an easily visualized graphical form [26], which has been widely used to identify the potential source of pollutants in the environment [27]. Concentrations obtained from chemical analysis for the 28 congeners were divided into 9 groups base on the chlorination levels, however, PCB 209 (CL-10) was not detected in any of the samples, thus 8 groups were used for the analysis. Individual congeners that were below the estimated analytical detection limit were set at one-half the detection limit. The eigenvalue for the first three principal components (PCs) accounted for 66% of the total variance of the 8 groups. Component 1 (PC1) was characterized by high chlorinated homologues (hexa-CBs, hepta-CBs and octa-CBs), whereas component 2 (PC2) was characterized by low chlorinated congeners (di-CBs, tri-CBs, tetra-CBs and penta-CBs), and component 3 (PC3) was characterized as nona-CBs. As shown in Fig. 5, it is possible to observe the presence of clusters in all samples based on the PCB chlorination level. The principal component plot indicated that the compositions of PCBs in most of the soil samples were similar, which probably originated from the same source. Among the 111 available soil samples, 106 of them could be classified into one group that was dominated by tetra-, hexa- and penta-CBs.

### Cancer Risk Assessment

Human risk assessment was evaluated via inhalation of soil particles, dermal contact and ingestion according to the equations described in *2.6*. Taking into the low level of PCB concentrations in this study, the results were only used to assess the potential impact of these measured PCBs on humans. As shown in Table 2, the risks of PCBs of different exposure pathway are far below 10^−6^, suggesting negligible adverse effects of those compounds. The total cancer risk is at the low cancer risks rank even at the 95^th^ percentile according to ATSDR standard. For different exposure pathways, the increasing trend in risks of cancer for PCBs was as follows: inhalation<dermal contact<ingestion.

**Table 2:** Total cancer risks (CR) from exposure via ingestion, dermal contact and inhalation of soil in humans on the total PCBs concentrations at the 5^th^ percentiles, median and 95^th^ percentiles.

CR via Exposure pathways	5^th^ percentile	Median	95^th^ percentile
Ingestion	0.019	0.11	0.34
Dermal contact	0.012	0.071	0.23
Inhalation	0.000042	0.00025	0.00079
Total	0.031	0.18	0.57

Note: The value of cancer risks are in the unit of 10^−6^.

### Conclusion

PCBs residual levels in the soils of the Midway Atoll were investigated. The results show that the concentrations of PCBs in this area were at the medium level compared with several different places around the world. According to the principal component analysis, the compositions of PCBs in most of the soil samples were similar, which indicated that the pollution of PCBs probably originated from the same source. Furthermore, risk assessments via ingestion, dermal and inhalation were evaluated based on the data obtained. The cancer risk of PCBs in the soils fell into very low range. It is noteworthy that Midway Atoll is contaminated with a number of pollutants such as PAHs and heavy metals. This study focused on PCBs cancer risk assessment. A comprehensive risk assessment is required for all significant pollutants for the area.
